# Reconnecting Brain Networks After Stroke: A Scoping Review of Conventional, Neuromodulatory, and Feedback-Driven Rehabilitation Approaches

**DOI:** 10.3390/brainsci15111217

**Published:** 2025-11-12

**Authors:** Jan A. Kuipers, Norman H. Hoffman, Frederick Robert Carrick, Monèm Jemni

**Affiliations:** 1 The Carrick Institute, Cape Canaveral, FL 32920, USA; drnorm@hoffmanwellness.com (N.H.H.); drfrcarrick@post.harvard.edu (F.R.C.); monemj@hotmail.com (M.J.); 2 Centre for Mental Health Research, University of Cambridge, Cambridge CB2 1TN, UK; 3 College of Medicine, University of Central Florida, Orlando, FL 32827, USA; 4 Burnett School of Biomedical Science, University of Central Florida, Orlando, FL 32827, USA; 5 MGH Institute for Health Professions, Boston, MA 02129, USA; 6 Faculty of Physical Education, Ningbo University, Ningbo 315000, China

**Keywords:** stroke rehabilitation, functional connectivity, neuromodulation, brain-computer interface, motor recovery

## Abstract

Background: Stroke leads to lasting disability by disrupting the connectivity of functional brain networks. Although several rehabilitation methods are promising, our full understanding of how these strategies restore network function is still limited. Here, we map how non-invasive brain stimulation (NIBS), brain–computer interface (BCI)/neurofeedback, virtual reality (VR), and robot-assisted therapy restore connectivity within the sensorimotor network (SMN), default mode network (DMN), and salience network, and we contextualize these effects within the known temporal evolution of post-stroke motor network reorganization. Methods: This scoping review adhered to PRISMA guidelines and searched PubMed, Cochrane, and Medline from January 2015 to January 2025 for clinical trials focused on stroke rehabilitation with functional connectivity outcomes. Included studies used conventional therapy, neuromodulation, or feedback-based interventions. Results: Twenty-three studies fulfilled the inclusion criteria, covering interventions like robotic training, transcranial stimulation (tDCS/TMS), brain–computer interfaces, virtual reality, and cognitive training. Motor impairments were linked to disrupted interhemispheric sensorimotor connectivity, while cognitive issues reflected changes in frontoparietal and default mode networks. Combining neuromodulation with feedback-based methods showed better network recovery than standard therapy alone, with clinical improvements closely associated with connectivity alterations. Conclusions: Effective stroke rehabilitation depends on targeting specific disrupted networks through various modalities. Robotic interventions focus on restoring structural motor pathways, feedback-enhanced methods improve temporal synchronization, and cognitive training aims to enhance higher-order network integration. Future research should work toward standardizing connectivity assessment protocols and conducting multicenter trials. This will help develop evidence-based, network-focused rehabilitation guidelines that effectively translate mechanistic insights into personalized clinical treatments.

## 1. Introduction

Stroke is a primary cause of long-term disability, impacting millions and resulting in notable motor, cognitive, and sensory impairments [1,2]. These impairments stem from disruptions in brain connectivity, which hinder communication between essential neural networks responsible for coordinated actions and cognitive functions [3,4]. Functional connectivity refers to the synchronized activity between brain regions that supports the execution of complex tasks. Following a stroke, disconnection of networks—particularly the motor, default mode, and salience networks—significantly limits recovery prospects [5,6].

**Figure 1:**
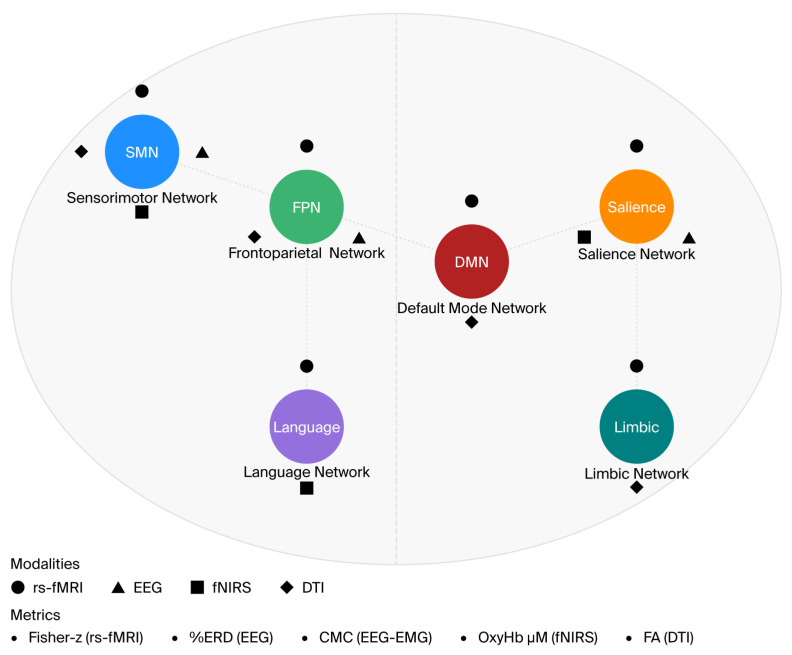
Network atlas and modality overlays.

The motor network, which is crucial for voluntary movements, is often disrupted by stroke-related damage, leading to impaired motor control and coordination [7,8]. Similarly, the default mode network (DMN), responsible for self-referential thought and cognitive processing, often shows altered connectivity in stroke survivors, contributing to deficits in memory and attention [9,10]. The salience network, which plays a key role in processing and prioritizing environmental stimuli, is also frequently affected, leading to difficulties in adapting to changing demands [11,12]. These disruptions highlight the importance of functional connectivity in post-stroke recovery and underscore the need for targeted interventions to restore network synchronization. Other systems—language, fronto-parietal control (FPN), and limbic–hippocampal circuits—are also disrupted after stroke. However, across 2015–2025 clinical trials reporting connectivity outcomes, the most reproducible mechanistic links to functional recovery involved the sensory motor network SMN, salience, and DMN; we therefore prioritized these networks for synthesis while highlighting the others as key targets for future connectivity-based rehabilitation. Figure 1 illustrates the core cortical–subcortical networks analyzed in the review (SMN, FPN, DMN, Salience, Language, Limbic).

Representative cortical–subcortical maps illustrating the principal networks analyzed across studies: sensorimotor (SMN; blue), frontoparietal (FPN; green), default mode (DMN; red), salience (orange), language (purple), and limbic/hippocampal (teal). Overlays indicate imaging modalities contributing connectivity data—rs-fMRI (circle icon), EEG (triangle), fNIRS (square), and DTI (diamond)—with legends identifying principal metrics (Fisher-*z*, %ERD, CMC, OxyHb μM, FA). The schematic summarizes multi-modal evidence linking network-specific coupling patterns to functional outcomes after stroke.

Advances in neuroimaging and electrophysiology have enhanced our understanding of post-stroke disconnection and its effects on outcomes [13,14]. Research indicates that rehabilitation techniques can help realign disrupted networks. Strategies like task-specific training and neurostimulation are promising for restoring connectivity and improving recovery. For example, task-specific therapies foster plasticity in motor networks, while non-invasive brain stimulation methods such as transcranial magnetic stimulation (TMS) and transcranial direct current stimulation (tDCS) support interhemispheric communication and network reorganization [15,16]. Recent studies involving brain–computer interfaces and neurofeedback offer innovative ways to modulate connectivity in real time, enabling personalized interventions that support recovery progress.

Post-stroke rehabilitation increasingly aims to restore functional connectivity to boost neuroplasticity and support motor recovery. Neuromodulatory tools like tDCS, TMS, and brain–computer interfaces (BCIs) are promising for influencing cortical activity and improving motor results. Traditional physical rehab remains widely used, though it often lacks real-time feedback. Integrating advanced feedback methods, such as quantitative electroencephalography (qEEG), with standard therapy could enhance recovery by directly targeting neural pathways underlying functional connectivity.

tDCS has been shown to improve motor learning and increase total coherence (TotCoh), indicating better brain synchronization after stroke [17,18]. It is especially effective in upper limb rehabilitation when combined with functional electrical stimulation (FES). In FES, precisely timed electrical pulses are applied to paralyzed or weakened muscles via surface electrodes placed over targeted muscles. These pulses cause muscle contractions aligned with the patient’s voluntary movements or brain–computer interface signals, aiding motor relearning and strengthening neural pathways [19]. Likewise, brain–computer interfaces (BCIs) use neural signals to provide real-time feedback, boosting motor recovery and cognitive functions [20,21]. Unlike conventional rehabilitation, which may not fully activate neuroplasticity due to limited neural reinforcement, these interventions harness neural mechanisms more effectively.

While neuromodulatory methods are gaining attention, physical rehabilitation remains crucial for stroke recovery. Its effectiveness can be improved by combining neurostimulation and feedback mechanisms [22,23]. This review discusses integrating feedback-based therapies with traditional physical therapy to find the most effective interventions for restoring functional connectivity after stroke.

Current rehabilitation methods, like traditional physical therapy, often have limitations in addressing neurobiological disruptions caused by stroke and neurological injury [24,25]. These include inadequate targeting of impaired cortical excitability, persistent neuroinflammation, and reduced neuroplasticity, all of which are crucial for optimal functional recovery [24,25,26]. Conventional therapies frequently result in plateaued motor gains, long-term compensatory movements, and incomplete motor function restoration [24,27]. Combining neuromodulatory interventions, such as repetitive transcranial magnetic stimulation (rTMS), with standard physical therapy helps address this clinical gap by encouraging neurophysiological adaptation in affected motor pathways. Neuromodulation enhances cortical plasticity and synaptic connectivity, thereby improving the effectiveness of traditional rehabilitation and enhancing motor and cognitive outcomes [24,25,28]. Reorganization after stroke typically progresses from early bilateral/contralesional recruitment toward later re-lateralization of ipsilesional motor pathways as recovery consolidates. Rehabilitation techniques likely interact with, and can accelerate, this trajectory by normalizing interhemispheric SMN coupling and restoring task-relevant oscillations [29].

This review hypothesizes that combining neurostimulation and feedback interventions with conventional stroke rehabilitation can restore functional connectivity. Disrupted neural networks—specifically the motor, default mode, and salience networks —can be reconnected to improve motor, cognitive, and sensory outcomes [30]. We assert that stroke-induced disconnections are a key factor in functional impairments and that targeted neuromodulatory strategies can boost neuroplasticity when paired with standard physical therapy.

To evaluate this hypothesis, the review addresses two primary research questions:

1. What are the specific patterns of functional connectivity disruption in the motor, default mode, and salience networks following a stroke, and how do these relate to different functional impairments?
2. Which rehabilitation protocols, including task-specific motor training, cognitive exercises, or neuromodulatory interventions, are most effective in enhancing network-specific connectivity and promoting functional recovery?

### Terminology

In this review, we define “functional reconnection” as a statistically significant increase in coupling between predefined nodes or edges within a network (for example, higher M1–M1 rs-fMRI Fisher-z values, stronger ERD symmetry, or increased CMC). We use “reorganization” to describe topological or redistribution changes in the network’s structure or dynamics—such as shifts in laterality indices, altered hubness or community assignments, or network-wide changes in path or synchrony metrics. This distinction helps clarify whether an intervention restores connection strength at specific edges or alters the overall network configuration.

## 2. Method

This review employs a scoping approach to capture the range of rehabilitative strategies for restoring network-level function after a stroke. We utilized the PRISMA framework to guide the literature search, selection, and data extraction. By focusing on interventions (robotic, neuromodulatory, and feedback-driven) and outcomes (motor, cognitive, vascular, and neuroimaging), a scoping review design ensures that heterogeneous or preliminary studies, which contribute to our understanding of post-stroke connectivity disruptions, are not excluded.

### Search Strategy and Selection Criteria

**Figure 2:**
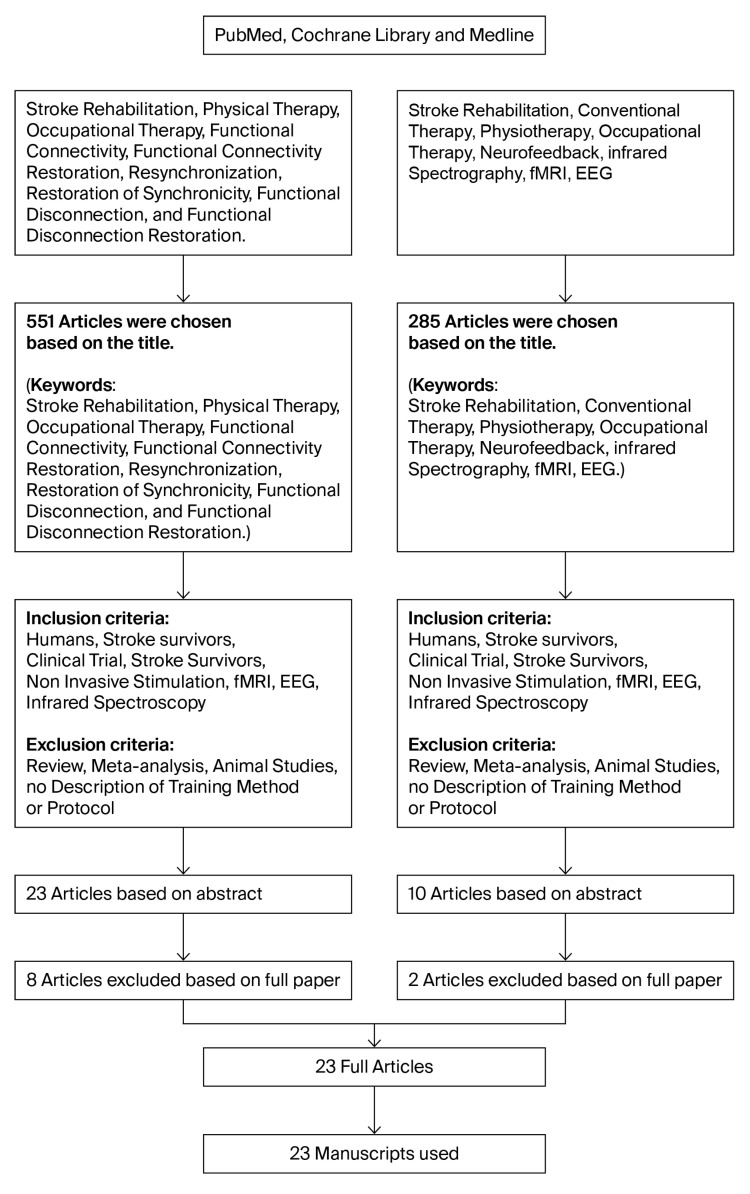
PRISMA-ScR flow diagram.

We searched PubMed, Cochrane, and Medline for articles published between January 2015 and January 2025, a ten-year period chosen to reflect recent advances in neuroimaging and neuromodulatory rehabilitation. After removing duplicates, two reviewers independently screened titles and abstracts, with a third reviewer resolving discrepancies. Full-text screening determined eligibility based on the criteria outlined below. The study selection process is summarized in Table 1. We followed PRISMA-ScR guidance for scoping reviews. The protocol was not prospectively registered (e.g., PROSPERO/OSF) given the exploratory aim to map heterogeneous, emerging connectivity outcomes; this is acknowledged as a limitation in §4.3. Reasons for exclusion at full-text review are provided in the PRISMA flow (Figure 2): no connectivity outcome, non-human study, review/meta-analysis, or no intervention.

A comprehensive literature search was performed to identify relevant studies on stroke rehabilitation and functional connectivity. An initial search focusing on stroke, connectivity, and therapy (including physical and occupational therapy, neurofeedback, fMRI, and EEG) yielded 551 articles. A second search, emphasizing conventional therapy, neurofeedback, and imaging, returned 285 results. Both sets were filtered to include only human studies and narrowed further by limiting to clinical trials and non-invasive protocols. Reviews, meta-analyses, animal studies, and articles without explicit protocols were excluded, reducing the first group to 23 abstracts and the second to 10. After full-text review, 15 and 8 articles, respectively, were considered suitable, totaling 23 articles. Additional refinements finalized the corpus for review.

A scoping review framework was selected because of the wide range of interventions (such as transcranial direct current stimulation, repetitive transcranial magnetic stimulation, robotic-assisted therapies, and brain–computer interfaces) and diverse outcome measures (including motor performance, executive function, imaging-based connectivity indices, and cerebrovascular parameters). This scope would be challenging to compile in a more restrictive systematic review or meta-analysis; however, a scoping approach enables mapping the evidence and pinpointing research gaps without excluding heterogeneous designs.

Two reviewers conducted a standardized data extraction using a structured form to record the study design, sample characteristics (including stroke type, sample size, and time since stroke), intervention details (frequency, intensity, and duration), and networks examined (motor, default mode, and salience). They also documented the methods used for connectivity assessment (fMRI, EEG, fNIRS), reported outcomes (motor and cognitive measures, clinical scales), and summarized key findings related to connectivity changes and recovery patterns. This information was then used to link specific patterns of network disruption to functional impairments and assess the effectiveness of interventions in restoring connectivity.

Both reviewers assessed methodological quality using validated appraisal tools to evaluate bias risk and study rigor. Disagreements were resolved through consensus. Due to the methodological diversity of the included studies, no quantitative meta-analysis or funnel-plot-based publication bias assessment was performed; instead, a qualitative synthesis identified common themes and notable gaps.

For randomized trials, we applied RoB 2; for non-randomized interventional studies, ROBINS-I. Two reviewers with consensus adjudication performed appraisals; per-study judgments appear in Table S1. We also provide a brief GRADE-style confidence statement for the main network-specific conclusions, recognizing the exploratory scoping design.

We aligned rs-fMRI correlation (Fisher-z), EEG oscillatory/synchrony indices (ERD/ERS, PLV/PLI, coherence, CMC), and fNIRS OxyHb coupling at the network level (e.g., M1–M1 within the SMN). Because many studies lacked variance parameters, we present (i) a vote-counting direction-of-effect summary and (ii) dose descriptors (intensity, minutes/session, sessions/week, total sessions). Where reported, we extracted standardized effects and confidence intervals.

Because of differences in study environments, some interventions took place in tightly controlled clinical trials with higher internal validity. Others were part of real-world rehabilitation programs with more varied patient populations, offering greater external validity. By recording treatment intensity, frequency, and patient adherence, we could compare how these factors affected the recovery of functional connectivity. Subgroup analyses distinguished protocols conducted in strict laboratory conditions from more flexible, patient-centered, or home-based approaches, such as tele-rehabilitation. This helped clarify how dosage, feedback type, and concurrent interventions might differently influence connectivity outcomes. Overall, this approach provides a thorough and systematic overview of current knowledge on rehabilitative strategies aimed at restoring disrupted neural networks post-stroke, while also identifying areas for future research. The overall study identification and selection process is depicted in the PRISMA flow diagram (Figure 1).

## 3. Findings and Thematic Synthesis

**Figure 3:**
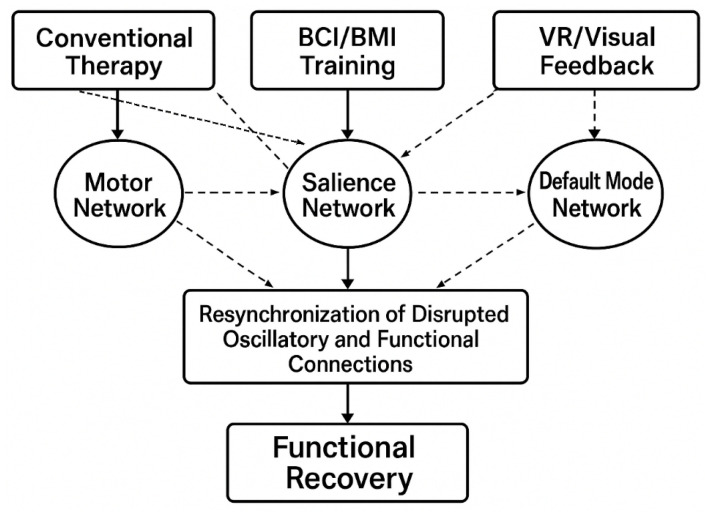
Conceptual Framework for Post-Stroke Network Resynchronization Through Rehabilitation Interventions.

This section offers insights synthesized solely from secondary sources, not new empirical data. Highlighting “Findings and Thematic Synthesis” emphasizes that the goal of the scoping review is to identify and interpret patterns, themes, and gaps across published studies, rather than to present original experimental findings. An overview of this framework is illustrated in Figure 3.

### 3.1. Quantitative Summary

Across the 23 included studies (Table 2), we extracted sample sizes, dose parameters (intensity; minutes/session; sessions/week; total sessions), and reported inferential statistics where available (t/F, p, confidence intervals, and effect sizes). Because statistical reporting was heterogeneous, we provide (i) descriptive statistics across studies (medians and interquartile ranges for study size and dose) and (ii) a vote-counting summary of direction-of-effect on connectivity (e.g., M1–M1 rs-fMRI Fisher-z, EEG ERD magnitude/symmetry, CMC, ALFF/ReHo) and clinical outcomes. Figures use consistent units; error bars denote 95% CIs for within-study metrics (or IQR for cross-study summaries). We did not impute missing variances and mark such entries as NR. Aggregate descriptive parameters for session intensity, duration, and sample size are summarized in Table 3. All extracted dose descriptors and study-level statistics are compiled in Supplementary Table S2, which extends Table 2 with quantitative parameters and greater transparency in reporting.

Units and Normalization Policy. To ensure comparability across modalities, we report: (i) rs-fMRI functional connectivity as Fisher-z transformed Pearson correlations; (ii) ERD/ERS as percent change from baseline (referenced to pre-task/baseline); (iii) synchrony indices (PLV/PLI/wPLI, coherence, PFCC) on a 0–1 scale (z-scored when aggregated across studies); (iv) ALFF/ReHo as z-standardized values; (v) fNIRS OxyHb in μM (or standardized units where concentrations were not reported), with acquisition/processing method described in the source; (vi) CMC as magnitude or Fisher-z, explicitly labeled; (vii) LI and BSI as unitless indices. Figure y-axes and table units follow this convention (e.g., “Fisher-z”, “%ERD”, “wPLI (0–1)”, “zALFF”, “OxyHb (μM)”).

Aggregated descriptive statistics across the 23 included interventional studies reporting quantitative dose parameters and participant characteristics. Median [IQR] values are derived from studies that explicitly reported numeric values; entries marked NR indicate missing or heterogeneous reporting. This summary supports the quantitative synthesis presented in Section 3.1.

**Figure 4:**
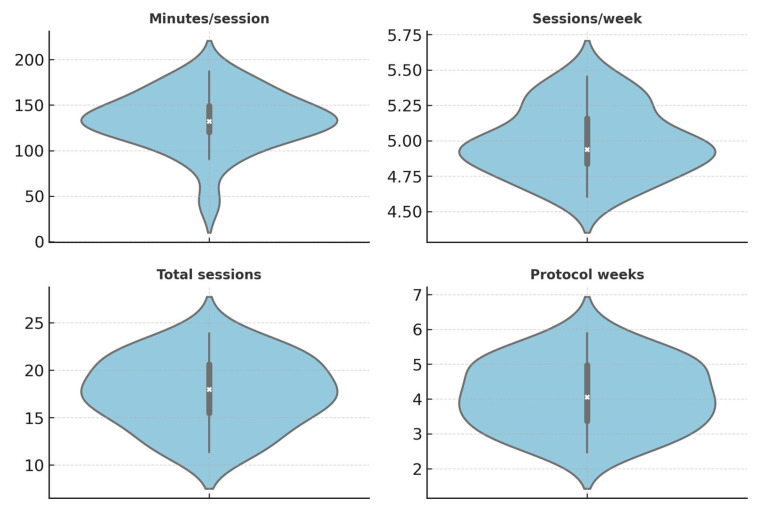
Dose distributions across studies. Box-and-violin plots display median ± IQR for reported minutes per session, sessions per week, total sessions, and protocol duration (weeks) from Table 2 and Supplementary Table S2. Horizontal dashed lines indicate global medians. Outliers represent highly intensive or shortened regimens, showing the variability of dosing across modalities (robotic, BCI/BMI, VR, NIBS, conventional).

Figure 4 shows the dose distributions across studies.

### 3.2. Cognitive Training

Cognitive deficits such as attention, memory, executive function, and spatial awareness often reduce the quality of life and independence of stroke survivors [54,55]. Recent advances in neuroscience emphasize the importance of functional connectivity—the temporal correlation between brain regions—as a crucial factor in cognitive recovery after stroke [7,56]. Therefore, cognitive training interventions that target neural plasticity and connectivity are becoming more popular as potential therapies to improve cognitive deficits following a stroke. Beyond tDCS/rTMS, transcranial alternating current stimulation (tACS) can entrain frequency-specific oscillations within cerebello-cortical and cortico-cortical pathways, thereby modulating functional connectivity. Hybrid protocols, such as γ-tACS combined with iTBS, can enhance and extend LTP-like plasticity in M1, indicating a promising synergy for rehabilitation dosing [57].

Disruptions in functional connectivity following a stroke often lead to changes in communication within major brain networks such as the default mode network (DMN), dorsal attention network (DAN), and executive control network (ECN). These alterations are directly linked to the severity of cognitive impairments seen in clinical settings [4,10]. fMRI research consistently indicates that improvements in cognitive functions through specific training are associated with the restoration or reorganization of these affected networks [5,58]. Notably, increased functional connectivity within cognitive networks after intervention may serve as a biomarker for cognitive recovery and treatment success [59,60].

Recent advances in cognitive training emphasize personalized methods that combine adaptive training, virtual reality, and brain stimulation techniques such as transcranial magnetic stimulation (TMS) and transcranial direct current stimulation (tDCS). These approaches have been demonstrated to improve functional connectivity and promote neuroplasticity [41,61]. Evidence indicates that integrating cognitive training with neurostimulation results in greater cognitive improvements compared to single-modality strategies, highlighting the importance of comprehensive therapeutic protocols [31,62]. The included studies and their connectivity-related outcomes are summarized in Table 2.

To understand how these interventions restore function, it is essential first to examine the specific patterns of network disruption that underline post-stroke cognitive and motor deficits.

#### 3.2.1. Functional Disconnection Patterns Underlying Cognitive and Accompanying Physical Dysfunction

Analyzing functional disconnectivity patterns in chronic stroke patients provides insights into the neural mechanisms underlying impaired motor and cognitive functions. A key study by Ripollés et al. highlighted disruptions in the auditory–motor network, showing decreased connectivity between the supplementary motor area (SMA) and the precentral gyrus (PRG) [52]. Chen et al. observed improved vasomotor reactivity in certain patient groups, suggesting partial recovery of connectivity, though the exact network effects remain unclear [50]. Gangemi et al. supported these findings with EEG assessments, revealing reduced activation in motor areas before therapy [44]. Although initial tests showed similar auditory activation to healthy controls, there was a marked decrease in activity in the affected hemisphere’s precentral gyrus and bilateral SMA, confirming significant auditory-motor disconnection consistent with Ripollés et al. The studies consistently show disrupted connectivity, especially in auditory-motor pathways, emphasizing its role in chronic stroke. Increased bilateral motor network activity indicates compensatory mechanisms due to ipsilesional disconnections [52]. Furthermore, impaired interhemispheric connectivity in motor regions highlights extensive neural disruptions in chronic stroke [52]. Persistent cerebrovascular changes reported by Chen et al. point to vascular factors in these disconnectivity patterns [50]. Recognizing these disruptions allows exploration of how cognitive training can promote network restoration and recovery.

#### 3.2.2. Connectivity and Synchronization Mechanisms Underpinning Cognitive Training Intervention Outcomes

Connectivity and synchronization mechanisms are increasingly understood as essential to evaluating the success of cognitive training in chronic stroke rehab. Music-supported therapy (MST), as studied by Ripollés et al., vividly shows how auditory–motor synchronization can be restored, reinstating functional connectivity within the SMA–PRG loop, normalizing disrupted activation, and promoting bilateral motor activity [52]. Similarly, Chen et al. indirectly supported network synchronization through combined computer-aided cognitive training (CACT) and transcranial direct current stimulation (tDCS) [53]. Although they did not directly measure functional connectivity, improvements in MoCA scores and BHI suggest vascular and cortical contributions to network recovery. They hypothesized that bilateral prefrontal stimulation via tDCS could enhance oxygenation and diffusion, accelerating functional healing. These studies underscore how targeted cognitive or neuromodulatory treatments can foster synchrony at cortical and subcortical levels. Gangemi et al. further explored VR cognitive training using EEG to track connectivity changes; following VR therapy, patients showed increased alpha and beta power—indicative of better attention, cognitive resource use, and motor planning—with alpha elevations in occipital areas and beta increases frontally, suggesting improved regional synchronization [44]. Gangemi et al. argued that VR-based interventions promote neurocognitive and motor restructuring by modifying connectivity across multiple networks. Collectively, these studies point to recurring mechanisms—such as enhanced auditory-motor coupling [44,52] and increased frontal activation and connectivity [44,50,52]—which underpin post-stroke cognitive and motor recoveries. The role of vascular health, highlighted by Chen et al., adds another layer of importance to connectivity restoration [50]. Overall, these insights confirm that neuroplastic reorganization supports the success of various cognitive rehab methods by restoring network functions, advancing our comprehension of how different interventions work at the network level.

### 3.3. Conventional Therapy

Despite ongoing technological advances, traditional therapies remain the main rehabilitation method because of their practicality and proven effectiveness [27,63].

Recent advances in neuroimaging have enhanced understanding of the neural mechanisms involved in recovery through traditional rehabilitation. Functional connectivity, an important biomarker, reflects the effectiveness of these interventions [7,12]. Conventional therapy encourages adaptive changes in functional connectivity within brain networks related to motor and cognitive functions, improving recovery outcomes [64,65].

Repetitive practice in traditional rehabilitation promotes reorganization within motor regions and interconnected networks, positively affecting motor functions [8,66]. Likewise, cognitive rehabilitation exercises can modify functional connectivity in attentional networks, leading to cognitive gains after stroke [10,67]. Research frequently investigates the mechanisms behind conventional therapy, which vary greatly depending on clinical settings and available patient resources [27,63]. In many cases, task-specific practice remains essential: therapists engage patients in daily activities like reaching or sit-to-stand transitions to promote meaningful recovery [22,68]. For example, inpatient programs might include 45-min sessions targeting repetitive motor tasks, assessed using tools such as the Functional Independence Measure (FIM) [69] and the Berg Balance Scale [70]. Correlating improvements in these assessments with neuroimaging can reveal links between recovery progress and brain coherence changes.

Home-based rehabilitation may also include scheduled therapist visits and customized exercise plans [71,72]. Patients might perform arm or balance exercises in front of a mirror, with weekly check-ins for support, especially in areas with limited resources [27]. Some clinics incorporate telehealth and wearable sensors for real-time monitoring of therapy intensity [72].

Case studies demonstrate the effectiveness of traditional therapy approaches. For example, a 62-year-old stroke patient might undergo six weeks of constraint-induced movement therapy (CIMT) combined with task-specific training, leading to improvements in Fugl-Meyer scores and increased motor cortex synchronization [73,74]. Another patient could focus on postural stabilization and gait training with the assistance of balance aids [75]. These routines can promote neuroplasticity, boosting neural connectivity, as shown in periodic assessments.

These examples highlight how structured exercises and goal-oriented tasks are essential for reconnecting disrupted networks [24]. Therapists modify protocols—whether Bobath [76], neurodevelopmental techniques, or resistance exercises—based on each patient’s functional level and environment. Although less advanced than virtual reality or brain–computer interfaces, conventional methods are broadly accessible and can incorporate connectivity principles into stroke rehabilitation. To understand conventional therapies, it is important to examine the specific network disruptions they aim to address.

#### 3.3.1. Functional Disconnection Patterns Underlying Sensory, Motor, and Cognitive Dysfunction

Post-stroke motor and sensory deficits are seen as disruptions in key brain regions’ functional connectivity. The disconnection between parietal and frontocentral areas is strongly linked to motor impairments. This connectivity change appears during movement-related cortical potentials (MRCPs) in two-legged gait tasks, indicating a breakdown in the timing needed for effective sensorimotor integration. Specifically, the parietal cortex, crucial for motor planning, fails to synchronize well with frontal premotor areas responsible for movement execution [42].

Specific motor-related disconnection patterns have been identified. Stroke often causes an imbalance between hemispheres, with increased alpha-band synchronization in the ipsilesional motor cortex and decreased activity in the contralesional hemisphere. These changes, especially in the motor cortices and cerebellum, are positively linked to motor recovery, highlighting the importance of restoring hemispheric balance for functional gains [42]. Corticomuscular coherence (CMC), which reflects the connection between the motor cortex and muscle activity, is another critical factor affected. Stroke-related reductions in CMC could serve as biomarkers for motor impairments and recovery prospects. Recent studies support using CMC measurements in brain–computer interface (BCI) systems to monitor recovery and guide targeted therapy. Additionally, a new sequential learning model utilizing graph neural networks (GNNs) has been created to combine EEG and EMG signals, enabling the detection of complex spatiotemporal patterns for personalized rehabilitation [43].

Furthermore, disrupted cerebellar–cortical circuits, especially those involving the cerebellar vermis, are linked to balance and postural control deficits. The vermis coordinates muscles crucial for stability, and impaired communication with cortical areas can hinder recovery [48,77]. These findings highlight the importance of focusing on cerebellar connectivity in interventions for balance and gait training.

Together, these insights highlight that stroke is not just a lesion-based disorder but also involves extensive functional disconnectivity. Connectivity studies show that focal lesions lead to changes in remote network activity, and abnormal interactions among motor regions are linked to impaired behavior and recovery prospects [7].

Rehabilitation strategies that include this connectivity perspective—such as targeted gait training for fronto-central–parietal synchrony restoration [7], neuromodulation targeting cerebellar pathways [2], and advanced EEG–EMG integrated systems for decoding movement intent [7]—may offer more effective, personalized approaches to recovery after stroke. Recognizing these disruption patterns helps us examine how traditional rehabilitation interventions restore affected neural pathways through connectivity and synchronization mechanisms.

#### 3.3.2. Connectivity and Synchronization Mechanisms Underpinning Conventional Intervention Outcomes

Recent studies emphasize the crucial role of neural connectivity in the success of traditional stroke rehabilitation. Parietal-frontocentral connectivity (PFCC) links sensory integration regions with motor execution areas and is synchronized with motor-related cortical potentials (MRCPs) during lower limb tasks in post-stroke patients. The timing of PFCC activation highlights its important role in planning and executing motor tasks. Variations in PFCC strength are linked to lower limb performance, indicating that improving this connectivity could lead to better functional recovery [42].

Corticomuscular coherence (CMC) measures the connection between motor cortex activity and muscle output, offering insights into brain-muscle interactions during voluntary movement. Recognized as a dynamic biomarker, CMC monitors motor recovery and cortical involvement in rehabilitation processes. Recent advances allow for precise modeling of these interactions, such as edge concatenation methods using symmetric partial mutual information (SPMI), providing a detailed analysis of corticomuscular communication. These methods quantify recovery progress and identify functional deficits, guiding targeted interventions [78].

Stimulating the cerebellar vermis is increasingly being explored for restoring posture and balance. Chen et al. investigated cerebellar vermis iTBS to activate the trunk and lower limbs, hypothesizing it modulates supplementary motor areas via cerebellospinal pathways [48]. These methods complement gait-focused therapies and may help improve balance and trunk control by influencing subcortical and cortical pathways. Ongoing research will reveal if combining cerebellar stimulation with traditional exercises produces better motor recovery. A promising advancement involves integrating EEG and EMG to analyze brain-muscle connectivity during rehab. This multimodal technique captures cortical intent and muscle activity, offering deeper insights into motor processes. Additionally, a graph neural network (GNN) model encodes sequential movements, allowing phase-specific analysis of intent and output. This supports more interpretable evaluations of movement complexity and neurofeedback, potentially leading to more personalized rehab plans [43].

These findings highlight the importance of using connectivity and synchronization mechanisms to improve rehabilitation strategies. Strengthening PFCC may enhance planning, while CMC metrics act as biomarkers for therapy adjustments. Cerebellar stimulation shows potential for improving postural control, and EEG-EMG integration offers a framework for recovery assessment. This connectivity-based perspective signals a shift toward individualized approaches in stroke rehabilitation, demonstrating how traditional therapies utilize neuroplasticity to improve functional connectivity and boost motor and cognitive outcomes.

### 3.4. Robot-Assisted Enhanced Conventional Therapy

Integrating robotic technologies in neurorehabilitation offers intensive, high-repetition therapy for individuals recovering from neurological injuries. Robot-Assisted Enhanced Conventional Therapy (RAECT) combines the consistency and accuracy of robotics with the adaptability of traditional therapy. This method aims to restore motor function and stimulate neuroplasticity essential for long-term recovery [79,80].

Robot-assisted therapies facilitate activity-dependent plasticity by guiding goal-oriented movements that impact cortical excitability and foster neural reorganization [81]. Neuroimaging and neurophysiological research demonstrate that these therapies activate dormant neural pathways, enhance ipsilesional corticospinal function, and reestablish the balance of interhemispheric inhibition [82,83]. Increasingly, recovery is seen as a process of restoring and strengthening existing neural networks rather than forming new ones [8,84].

RAECT can improve recovery by engaging network mechanisms, reactivating peri-infarct regions, recruiting secondary motor areas, and dynamically altering connectivity [85]. These changes happen through Hebbian learning, synaptic strengthening, and axonal sprouting, especially in high-intensity, feedback-rich settings provided by robotic systems. To understand how these interventions promote neuroplasticity, it is important to analyze the network disconnections they aim to address.

#### 3.4.1. Functional Disconnection Patterns Underlying Sensory, Motor, and Cognitive Dysfunction

Recent evidence indicates that integrating robotic systems with traditional therapy helps address disconnections by fostering network-level neuroplasticity [50,86]. Reduced interhemispheric connectivity is common in post-stroke hemiparesis [38]. Fan et al. discovered that patients showed decreased resting-state functional connectivity between ipsilesional and contralesional primary motor cortices (M1), which correlated with the severity of motor impairment [38]. Likewise, Wittenberg et al. reported asymmetrical intrahemispheric connectivity, with less synchrony in the lesioned hemisphere, indicating that stroke impacts broader motor networks [37]. Robotic assistance improves traditional rehabilitation by enabling high-intensity, task-specific, and repetitive movement training—key factors for experience-dependent plasticity [38,50]. When combined with conventional therapy, robotic systems enhance motor network reorganization [32,49]. For example, Fan et al. showed that four weeks of robot-assisted bilateral arm training increased M1–M1 functional connectivity, linked to improved upper limb function [38]. Wittenberg et al. found that after upper limb therapy, patients exhibited increased connectivity between affected M1 and areas like contralesional M1, suggesting recovery within a distributed sensorimotor network [37]. Multimodal methods confirm these neuroplastic improvements [50]. Cheng et al. used synchronized fNIRS and surface EMG to demonstrate that robot-assisted hand therapy activates motor-related cortical areas and enhances muscle output, supporting cortico-muscular integration [50]. Qin et al. showed that exoskeleton-assisted hand rehabilitation with fingertip haptic stimulation results in stronger sensorimotor cortex activation, emphasizing the importance of multisensory feedback in therapy [32].

Recovery also involves cognitive systems related to planning and control, especially in patients with severe impairments [47,49]. Ramos-Murguialday et al. demonstrated that BMI training combined with conventional therapy increased activation in the prefrontal and premotor cortices, indicating recruitment of higher-order resources during motor learning [49]. Carino-Escobar et al. found that beta-band EEG activity was linked to better motor outcomes, reflecting broader network engagement [47]. Khademi et al. showed that proprioceptive neurofeedback in the beta-band frequency modulated cortico-muscular control, supporting the idea that robotics and neurofeedback target motor and cognitive domains [87].

The intensity and repetition of task-specific practice promote neural reorganization, regardless of the type of therapy [37]. Wittenberg et al. found no significant differences in brain connectivity outcomes between robotic and traditional therapy, indicating similar plasticity mechanisms [37]. However, robotic systems improve therapy delivery through consistent movement guidance, real-time feedback, and adjustable intensity, providing advantages within traditional frameworks [32,38]. Ramos-Murguialday et al. highlighted that BMI training combined with standard physiotherapy resulted in superior motor gains compared to sham controls, supporting the potential of hybrid interventions [49]. Several studies examined whether changes in brain connectivity can predict treatment response [37,38]. Wittenberg et al. found that baseline connectivity between affected M1 and dorsal premotor cortex could predict functional outcomes [37]. Fan et al. showed that increases in M1–M1 connectivity mediated the relationship between therapy and motor recovery [38]. Cheng et al. also supported this by demonstrating that real-time neurophysiological data monitored cortical engagement and muscular response, suggesting avenues for personalized intervention strategies [50]. Overall, these findings demonstrate that robot-assisted conventional therapy restores functional connectivity and advances stroke recovery [38,49,50]. This approach facilitates the reorganization of sensorimotor and cognitive networks by combining the adaptive elements of conventional therapy with the precision and feedback of robotics [32,37]. Ongoing research may pave the way for personalized rehabilitation protocols that enhance recovery by tailoring interventions to neural responsiveness [47,87]. Recognizing these disconnection patterns allows exploration of how robot-assisted interventions can restore network connectivity and improve recovery.

#### 3.4.2. Connectivity and Synchronization Mechanisms Underpinning Robotics Enhanced Conventional Intervention Outcomes

Emerging evidence highlights the potential of robotics-enhanced interventions to improve functional recovery in stroke rehabilitation through network connectivity and cortico-muscular synchronization. These neuroplastic changes reinforce behavioral improvements, driven by robotic precision and task-specific engagement characteristic of conventional therapy.

Fan et al. studied how robot-assisted bilateral arm therapy (RBAT) affects brain connectivity in people with chronic stroke [38]. They found that RBAT notably boosts interhemispheric connectivity between the ipsilesional and contralesional primary motor cortices (M1–M1), which correlates with improved motor skills and functional ability [38]. Additionally, RBAT promotes connectivity across multiple brain regions, showing that robotics-based therapies can foster large-scale reorganization of motor and associative networks. Changes in M1–M1 connectivity significantly mediated therapy success and recovery, indicating that restoring communication between the motor cortices across hemispheres is key to achieving functional improvements [38].

Wittenberg et al. found that intensive upper limb rehabilitation, combining robotic and conventional therapy, improved synchronization between the affected M1 and various motor-related cortical areas [37]. They observed no significant differences in connectivity outcomes between the therapies, suggesting that the intensity and repetition in both methods may mainly drive reorganization.

Ramos-Murguialday et al. investigated how brain-machine interface (BMI) training impacts motor control networks in conjunction with physiotherapy [49]. Their findings showed that BMI training increased the influence of nonprimary motor areas, particularly the premotor cortex, on finger extensor muscles, indicating adaptive cortical changes. Additionally, they observed a positive link between increased beta-band cortico-muscular coherence and clinical progress, underscoring the importance of synchronization in recovery.

Qin et al. examined the impact of exoskeleton-assisted hand rehabilitation with fingertip haptic stimulation, finding significantly greater activation in M1, premotor cortex (PM), and primary somatosensory cortex (S1) compared to robot assistance alone [32]. This approach increased the amplitude of the P300 component, signifying enhanced attentional and sensory processing, which suggests improved cortical engagement [51].

Carino-Escobar et al. examined EEG beta-band activity during a BMI intervention using robotic hand orthoses in stroke patients [47]. Their results showed that beta-band activity in frontal, central, and parietal regions was associated with motor recovery, indicating the involvement of broader cortical networks as a compensatory mechanism.

These studies collectively demonstrate that robotics-augmented interventions stimulate neuroplasticity by boosting both interhemispheric and intrahemispheric connectivity, as well as cortico-muscular coherence. This facilitates motor execution, advanced sensorimotor integration, and cognitive-motor coordination. The uniformity of results across different therapies suggests that recovery results from synchronized cortical activity within functional networks, enabled by the repetitive, feedback-rich, task-focused nature of these methods. Therefore, robotics-supported therapy offers a promising approach for motor function recovery by addressing both structural and dynamic brain connectivity, utilizing technology and neuroplasticity to achieve better outcomes than traditional therapy alone.

### 3.5. BMI, BCI, Virtual Reality, Visual Feedback-Enhanced Conventional Therapy

Recent advances in neurorehabilitation include the use of non-invasive brain–computer interfaces (BCIs), brain-machine interfaces (BMIs), virtual reality (VR), and therapies that utilize visual feedback. When combined with traditional treatments, these modalities provide feedback-driven interventions that target motor, perceptual, attentional, and cognitive functions [32,47,49]. Their integration aligns with key neuroplasticity principles—such as repetition, task specificity, sensory salience, and goal-directed learning [81]—creating a multisensory environment that encourages adaptive network reorganization in individuals post-stroke [47,49]. Unlike robotic systems that focus on movement, BMI and BCI techniques prioritize neural engagement and decoding of motor intent, enabling patients with severe motor deficits to operate devices through brain activity [47,49]. Ramos-Murguialday et al. showed that BMI training combined with physiotherapy improved movement and increased activation in brain regions, especially the ipsilesional premotor cortex, indicating effective voluntary control recovery [49].

EEG-based BCIs also interpret motor intentions and offer feedback to support neuroplasticity. Carino-Escobar et al. observed that using a robotic hand in a BCI intervention increased beta-band activity in certain brain areas, suggesting the engagement of compensatory networks in patients with limited function [47].

Similarly, virtual reality (VR) rehabilitation boosts patient engagement by offering interactive simulations that provide rich sensory feedback [88]. VR creates an immersive sensory environment and allows for intensive task repetition, which enhances brain activity through goal-oriented feedback, thereby strengthening motor and cognitive pathways [89]. The sense of presence fostered by VR activates cognitive networks crucial for engagement and learning [90].

Visual feedback-enhanced therapies use traditional methods like visual mirrors and augmented displays to correct movement errors and aid motor execution by activating relevant brain pathways [91]. This method is similar to mirror therapy, which stimulates motor-related brain regions through observational learning [91,92].

Neurofeedback and virtual interventions depend on real-time synchronization of brain activity with sensory input and motor output. Using various tools, they boost error-based learning, which is crucial for neuroplasticity [93]. Their flexibility allows integration into a wide range of therapy protocols, helping patients with different impairments [49,88].

Overall, BMI, BCI, VR, and visual-feedback therapies are neuroadaptive interventions that actively reshape brain connectivity. Their effectiveness depends on integrating advanced technology with conventional motor learning methods [81], supporting motor recovery by harnessing the brain’s ability to reorganize through feedback-based experiences [43,47,49]. To fully understand these interventions, it is important to analyze the complex network disconnection patterns they aim to target. Unlike open-loop NIBS, VR/BCI/neurofeedback operate as closed-loop controllers in which neural intent (ERD/SMR) is contingently reinforced by proprioceptive/visual feedback. This temporal contingency drives Hebbian learning, restores phase coupling across motor hubs, and generalizes to behavior beyond passive stimulation.

#### 3.5.1. Functional Disconnection Patterns Underlying Sensory, Motor, and Cognitive Dysfunction

Several studies emphasize the disconnection between hemispheres in stroke survivors, especially those with severe motor impairments. Ramos-Murguialday et al. found that chronic stroke patients had decreased resting-state connectivity between primary motor cortices (M1) compared to healthy controls, linking this disconnection to motor deficits and targeting it in their BMI intervention, which linked motor intent with outcomes [49]. After therapy, M1–M1 connectivity increased and correlated with clinical recovery, highlighting the importance of restoring interhemispheric synchrony [49].

Sinha et al. observed that stroke patients undergoing BMI training with functional electrical stimulation exhibited intrahemispheric connectivity centered on the ipsilesional M1, which differs from the typical bilateral patterns seen in healthy individuals [35]. After the intervention, interhemispheric connectivity was enhanced and was associated with motor improvements. This indicates that stroke-related sensorimotor disconnection involves both structural and functional aspects, and technology-supported therapies can help restore cortical balance [35].

Functional disconnection impacts cognitive and attentional networks and is often overlooked in rehabilitation. VR-based cognitive therapies have been proven to boost cortical rhythms and connectivity in areas responsible for executive functions. Gangemi et al. found that stroke patients undergoing immersive VR training showed increased EEG alpha and beta power, unlike those in traditional therapy [44]. This suggests that VR might more effectively stimulate cognitive networks by providing multisensory immersion [44].

Phang et al. showed frontoparietal disconnection during motor imagery in stroke patients, which reduced BCI system performance [46]. Their findings indicate that disrupting the frontoparietal network impacts intention-driven interfaces, emphasizing the need for interventions to reactivate motor control regions.

Wada et al. developed a proprioceptive feedback-based BMI that uses event-related desynchronization (ERD) to interpret motor intentions [40]. This approach enhances synchronization between brain signals and sensory feedback, thereby improving motor learning. Such proprioceptive cues could also aid in restoring sensorimotor coupling after a stroke.

Ray et al. found that beta-band power serves as a biomarker for motor recovery, with patients exhibiting greater improvements showing unique EEG patterns during tasks [45]. This indicates that the return of rhythmic activity is linked to restored function, making it a promising target for neurofeedback and BMI-based rehabilitation.

Overall, research indicates that rehabilitation approaches using closed-loop feedback through BMI, BCI, VR, or visual augmentation more effectively activate disconnected neural networks than traditional therapies. Ramos-Murguialday et al. demonstrated that combining BMI with physiotherapy resulted in greater motor improvements and restored M1–M1 connectivity compared to physiotherapy alone [49]. Sinha et al. found that VR limb mirroring enhanced interhemispheric integration and patient outcomes beyond conventional therapy [35]. Additionally, Gangemi et al. observed that only VR training caused neuroplastic changes [44].

These results show that fixing functional disconnection by syncing motor intent and feedback is key for recovery. While traditional therapies are important, they might lack the neural precision needed for network reorganization. Innovations in BMI, VR, and neurofeedback effectively help restore disrupted networks, supporting coordinated movement and thinking. Once we identify these disconnection patterns, we can investigate how feedback-enhanced interventions restore connectivity and synchronization.

#### 3.5.2. Connectivity and Synchronization Mechanisms Underpinning Feedback-Enhanced Conventional Intervention Outcomes

Ray et al. identified that alpha band oscillatory activity, particularly event-related desynchronization (ERD) during motor imagery, serves as a dependable biomarker for motor recovery in chronic stroke patients [45]. Patients exhibiting higher alpha ERD at baseline and greater desynchronization during training experienced more notable functional gains. A shift toward desynchronization in the ipsilesional hemisphere associated with clinical improvements suggests that restoring hemispheric balance is crucial for recovery. Similarly, Wada et al. developed a BCI system that utilized mu band ERD (8–13 Hz) to activate proprioceptive feedback for hand movements within a virtual environment [40]. This feedback loop, synchronized with motor imagery, likely promoted sensory-motor pathway reorganization by increasing cortical activity and enhancing the coupling of sensory input.

Connectivity within frontoparietal networks plays a vital role in stroke rehabilitation success. Phang et al. found that lower alpha-band connectivity between frontal and parietal areas is linked to better motor preparation-based BCI control [46]. This indicates that adjusting or even decoupling activity in frontoparietal circuits can enhance BCI performance by supporting motor intention encoding. Notably, stroke patients showed reduced frontoparietal connectivity compared to healthy individuals during motor preparation and execution, implying widespread disruptions in top-down motor planning.

Sinha et al. examined a virtual reality limb mirroring approach for patients in the subacute stroke phase, observing a transition in functional connectivity from within a single hemisphere to bilateral engagement [35]. They found that stronger connectivity between the primary motor cortices on both sides was linked to better motor function. The authors suggested that visual stimulation of the mirror neuron system through limb mirroring promoted reorganization between hemispheres, leading to improved synchronization.

Supporting this, Ramos-Murguialday et al. demonstrated that BMI training with functional electrical stimulation improved interhemispheric M1–M1 connectivity, which was linked to better upper limb function [49]. fMRI revealed a temporary normalization of contralateral M1 activation during paretic hand movements after the intervention. Although this shift in lateralization was not sustained at the six-month follow-up, it highlights the importance of early cortical re-engagement in recovery. The BMI system used sensorimotor rhythm desynchronization to provide proprioceptive and visual feedback, creating a feedback loop that likely encouraged sensorimotor reconnection.

EEG asymmetry measures, such as the Brain Symmetry Index (BSI), show a significant correlation with motor function; lower BSI scores, indicating better symmetry, are linked to improved performance. The Laterality Coefficient (LC), which assesses ERD asymmetry, also relates to upper limb function, implying that symmetric activation of the hemispheres improves outcomes. Sinha et al. found that BCI training with functional electrical stimulation enhanced interhemispheric connectivity within the motor network, resulting in improvements across various motor domains [35]. This indicates that BCI feedback can reorganize the overall motor network and boost its connectivity, promoting better cortical communication both locally and across the brain.

In cognitive research, Gangemi et al. found that VR-based cognitive training for chronic stroke patients increased alpha power in occipital regions and beta power in frontal areas, suggesting improved functional connectivity within visual and executive control networks [44]. The use of real-time audiovisual feedback probably supported multisystem reorganization by promoting task-specific, feedback-enhanced engagement.

Across these studies, converging mechanisms become apparent. Restoring interhemispheric balance in motor regions is linked to better clinical outcomes. Event-related desynchronization in the alpha and mu bands serves as a neural marker of motor imagery engagement and promotes plasticity when combined with feedback. Additionally, multisensory feedback—whether visual, proprioceptive, or auditory—is essential for strengthening the connection between motor intention and execution. These findings suggest that feedback-enhanced interventions can effectively promote synchronization and plasticity in disrupted neural circuits, complementing traditional therapy in personalized, connectivity-focused stroke rehabilitation. This evidence indicates that feedback-based interventions improve therapeutic outcomes by restoring the temporal alignment of disrupted neural networks through closed-loop mechanisms.

## 4. Discussion

This scoping review systematically addresses the two primary research questions posed in the introduction, offering critical insights into post-stroke network disruption and the efficacy of rehabilitation.

### 4.1. Mechanisms of Network Restoration

#### 4.1.1. Addressing Research Question 1: Patterns of Functional Connectivity Disruption

Our analysis revealed distinct network-specific disruption patterns that correlate with particular functional impairments. In the motor domain, impaired interhemispheric communication—especially between primary motor cortices (M1–M1)—along with weakened intrahemispheric pathways, such as parietal–premotor coupling during gait and cortico-muscular coherence, represent the core neural substrates of persistent motor deficits [38,42]. Cognitive dysfunction arises from breakdowns in the auditory–motor loop (SMA–PRG, PAC–IFG) that disconnect sensory feedback from motor planning, intensifying perceptual challenges [44,52]. While early evidence indicates that the frontoparietal, default mode, and salience networks are involved in post-stroke cognitive impairments, their precise contributions to functional deficits remain unclear.

#### 4.1.2. Addressing Research Question 2: Rehabilitation Protocol Effectiveness

Our results indicate that different rehabilitation approaches promote network-specific recovery through unique mechanisms. Traditional high-repetition practices encourage gradual reorganization of the motor network, which is further enhanced by neuromodulation techniques like cerebellar iTBS or multimodal EEG–EMG feedback [48,66]. Robot-assisted therapies offer the intensity and precision needed for strong M1–M1 recoupling and the recruitment of perilesional areas by restoring structural connectivity [50]. Feedback-based methods—including BMI/BCI systems, immersive virtual reality, and visual feedback—use real-time brain–machine interaction to restore functional connectivity by precisely timing neural networks [35,49]. Cognitive training, from music-supported therapy to tDCS-enhanced exercises, normalizes auditory–motor and prefrontal connections, improving both cognitive abilities and vascular responsiveness [52,53].

#### 4.1.3. Key Finding: Modality-Specific Network Targeting

The evidence indicates that no single intervention can effectively target all disrupted networks. Instead, successful rehabilitation involves a strategic combination of approaches: robotic interventions are particularly good at repairing structural motor pathways, feedback-enhanced methods help restore functional timing and synchronization, and cognitive training focuses on higher-level network integration. This approach aligns with precision medicine, in which individual connectivity profiles and specific network deficits inform the selection of interventions. Improvements in interhemispheric SMN coupling (M1–M1), task-related mu/alpha ERD, and β-band CMC imply that rehabilitation initially restores interhemispheric competition and later reinforces ipsilesional lateralization as skills develop. The salience network facilitates switching between DMN and FPN during tasks, explaining why closed-loop VR/BCI/neurofeedback—which enhances salience and prediction-error signals—yields stronger resynchronization than open-loop stimulation. Early contralesional activation suggests compensatory mechanisms or diaschisis, while sustained recovery depends on re-lateralization and peri-lesional integration. Cerebello-thalamo-cortical circuits likely initiate cortical timing and support Hebbian plasticity during closed-loop exercises.

#### 4.1.4. Integrated Framework for Clinical Translation

To guide clinical translation, we integrate patient phenotype (“who”), network targets (“where”), session timing/dose (“when”), and monitoring biomarkers into a unified actionable framework: For subacute (<3 months) subcortical or peri-M1 presentations, use bi-hemispheric tDCS or iTBS as priming, followed immediately by high-dose, feedback-rich, task-specific, and/or robot-assisted practice to enhance interhemispheric M1–M1 and parietal–premotor coupling [15,16,28,30,37,38,42,55,79,80]. For chronic (>6 months) cases with ongoing interhemispheric imbalance, prioritize closed-loop BCI/VR/neurofeedback that links ERD/SMR-based intent to contingent proprioceptive/visual feedback to restore timing-dependent synchrony and improve corticomuscular coherence (CMC) [35,40,45,49,87,88]. Targeting is network-specific: SMN (M1–SMA) → NIBS priming combined with feedback-rich practice [7,8,13,21]; frontoparietal control → incorporate immersive VR and action-observation therapy to boost top-down engagement [44,46,88,89,93]; default-mode/salience dysregulation → deploy CACT (computer-assisted cognitive training) with optional prefrontal NIBS as adjunct [31,53,74]. Timing and dose: administer NIBS before practice to leverage short-term plasticity windows, and ensure at least about 15 h of feedback-rich practice over three or more weeks, aligning with intensity-dependent gains [16,21,27,28,69,79,80]. Biomarkers: higher α/μ-band ERD during action observation or motor imagery indicates better recovery prospects, while increases in interhemispheric M1–M1 rsFC and CMC reflect network normalization and functional improvement during therapy [12,36,38,40,45,49,61,87,93]. See Table 4 for network guided timing and dosage.

This table summarizes clinically actionable timing and dosing recommendations for network-guided post-stroke rehabilitation. It integrates patient phase, dominant network disruption, recommended intervention pairings, and associated neurophysiological biomarkers.

##### Confounders

Understanding changes in connectivity after a stroke requires careful management and documentation of various bias sources. Biological and clinical differences—such as lesion location and size, chronicity, initial severity, and concurrent therapies or medications—can significantly impact network metrics and recovery patterns [5,7,14]. Additionally, modality-specific artifacts matter: head movements and processing choices in fMRI; volume conduction and reference selection in EEG; and systemic physiological effects in fNIRS [13,18,50]. Task design and delivery introduce further variability through difficulty levels, engagement, and factors related to therapists and the environment. Decisions made at the device level in BCI/BMI pipelines (like calibration, thresholding, or classifier updates) can also alter neural readings independently of actual recovery [35,40,46,49]. To improve trial comparability, we recommend transparent dose reporting, standardized task-difficulty measures, and data-quality metrics based on motor-learning principles and intensity guidelines [21,22,69,84].

##### Broader Implications

A network-guided method enhances pragmatic clinical decision-making by categorizing patients according to their connectivity phenotype. It combines priming neuromodulation with feedback-rich, task-specific exercises (hybrid dosing) and broadens access through tele-rehabilitation supported by analytics [7,16,17,66,72,73]. In practice, the real-time monitoring of event-related desynchronization (ERD), cortico-muscular coherence (CMC), and interhemispheric M1–M1 coupling, alongside behavioral assessments, enables immediate adjustments and may shorten the time to see benefits [12,36,38,40,45,87]. These closed-loop, precision approaches are consistent with evidence indicating that targeted stimulation and high-dose practice correct abnormal coupling and oscillations in sensorimotor and control networks, leading to mechanistic improvements that translate into functional gains [15,16,17,35,49]. Key connectivity measures used to quantify normalization and recovery are summarized in Table 5.

Summary of the most frequently reported connectivity metrics in post-stroke rehabilitation studies, their operational definitions, functional relevance, normalization schemes, and main interpretive considerations. This table provides standardized terminology for metrics referenced in Section 3 and Section 4.

### 4.2. Clinical Translation

Translating network-based rehabilitation insights into clinical practice offers both promising opportunities and notable challenges. Robot-assisted therapies are the most widely adopted, with well-established protocols for restoring M1–M1 connectivity that lead to observable functional improvements within 4–6 weeks [37,38]. These interventions’ dose-dependent nature aligns with current rehabilitation standards, easing their integration into routine care. While feedback-enhanced modalities deliver higher precision in synchronizing the temporal network, they require specialized technical infrastructure and training. BMI/BCI systems that restore cortico-muscular coherence are effective clinically, but their deployment demands significant equipment and expertise [49]. Virtual reality therapies serve as a balanced option, offering immersive feedback environments that are adaptable across clinical settings without losing therapeutic accuracy [35,44]. Cognitive training methods, particularly music-supported therapy and tDCS-augmented protocols, hold immediate clinical promise because they can be implemented in various settings with moderate resources [52,53]. Combining traditional therapy with targeted neuromodulation provides a practical approach for embedding connectivity-based interventions into current rehabilitation frameworks.

### 4.3. Limitations of Current Evidence

Despite progress, turning mechanistic insights into routine clinical practice remains challenging due to methodological constraints. Researchers utilize a range of imaging and electrophysiological methods—such as fMRI, EEG, and fNIRS—with unique analytical approaches, making comparisons and data integration difficult. A major obstacle is the inconsistent definition and measurement of “functional connectivity” across these modalities: fMRI assesses BOLD signal correlations, EEG examines oscillatory coherence, and fNIRS measures hemodynamic coupling. This variability hampers efforts to develop standardized connectivity biomarkers useful for clinical decisions.

Small, single-center studies with limited randomization reduce statistical reliability and validity, especially for emerging treatments like RAECT and closed-loop neurofeedback. Additionally, the differences in stroke features, lesion sites, and timing after injury make it harder to apply findings broadly across diverse patient groups.

Limited assessment of the default mode and salience networks leaves gaps in understanding cognitive and emotional outcomes. Although motor network recovery has been explored, the contribution of higher-order cognitive networks is not well understood, restricting the development of comprehensive rehabilitation approaches.

Furthermore, the absence of long-term follow-up data limits insights into whether connectivity improvements and independence are maintained over time. Most research assesses results only weeks or months after treatment, so the long-term stability of network changes and their impact on quality of life remain unclear.

Economic barriers also hinder implementation because high costs, technical complexities, and the need for specialized training for robotics and neurofeedback systems limit their widespread use, especially in resource-limited settings.

### 4.4. Future Research Trajectories

To develop a precision-connectivity approach in stroke rehab, future research needs to overcome current limitations by following strategic steps. Standardizing methods should involve creating consensus protocols for data collection and analysis, allowing studies to be directly compared and supporting meta-analyses. Large, multicenter trials that classify patients by lesion location and severity will help confirm the effectiveness of treatments across different groups and aid in developing evidence-based, connectivity-focused rehab guidelines. These should include standardized outcome measures that accurately reflect improvements in network function and overall recovery.

Expanding assessments to include default mode and salience circuitry will clarify their roles in cognitive recovery and guide therapeutic strategies. Understanding how cognitive and motor networks interact during rehabilitation will support the development of comprehensive intervention methods.

Longitudinal studies lasting six to twelve months after intervention are crucial for assessing the durability of neural reorganization and its effects on everyday living. These studies should investigate how ongoing connectivity modifications are connected to long-term independence, quality of life, and caregiver strain.

Future protocols ought to utilize baseline connectivity profiling to guide intervention choices, dosing, and sequencing. Machine learning can detect connectivity signatures that forecast treatment response, enabling personalized rehabilitation tailored to each person’s neural architecture.

The creation of portable feedback platforms that include EEG, EMG, and fNIRS within intuitive virtual environments can make advanced treatments more accessible, enabling home-based and remote rehabilitation. These technologies should focus on ease of use and affordability to overcome barriers to access.

Targeted training programs that address economic and infrastructure barriers will improve clinical adoption. Implementation research should investigate strategies for integrating connectivity-based interventions into healthcare systems, ensuring they are both practical and cost-efficient.

By integrating detailed mechanistic research with scalable, patient-centered care models, the field can transition from proof-of-concept studies to sustainable strategies that restore network integrity and enhance the recovery potential of stroke survivors. Evidence supports a future in which rehabilitation protocols are tailored to address functional deficits and the specific patterns of network disconnection underlying each patient’s impairments.

### 4.5. Limitations—Scope and Analytical Gaps

This scoping review was not pre-registered; thus, we clarify the reasons for exclusions in the PRISMA flow diagram (Figure 1) and present our findings as hypothesis-generating. Our synthesis highlighted motor, default-mode, and salience networks, but higher-order systems—such as language, frontoparietal control, and limbic–hippocampal circuits—were not thoroughly examined and should be prioritized in future research [3,4,5,10,51]. Notably, among the included interventional studies, no effective-connectivity methods (e.g., DCM, Granger causality) were used, and no consistent reporting of graph-theoretical predictors was observed, despite their relevance for understanding network pathology and rehabilitation [6,13,66].

## 5. Conclusions

This scoping review offers a comprehensive overview of how disruptions at the network level contribute to motor, sensory, and cognitive impairments following a stroke. It critically assesses the effectiveness of current rehabilitation methods in restoring network integrity. Analyzing 22 clinical studies published between 2015 and 2025, this analysis uncovers four key insights:

First, stroke should be viewed primarily as a disorder of large-scale brain network integrity rather than solely as focal tissue damage. Throughout both acute and chronic stages, persistent deficits are most consistently associated with weakened inter- and intra-hemispheric sensorimotor connectivity (such as M1–M1 coupling, parietal–premotor interactions, corticospinal coherence), dys-synchrony in higher-order cognitive networks (like default-mode, fronto-parietal, and salience networks), and impaired cerebrovascular reactivity, which further disrupts functional connectivity. These network issues go beyond lesion location and timing, highlighting the need to include network-level assessments in both diagnosis and treatment planning.

Second, different intervention strategies target various aspects of network dysfunction. High-dose, task-specific practice gradually retrains residual corticospinal pathways, while robot-assisted therapy speeds up the structural reconnection of bilateral sensorimotor hubs. Non-invasive neuromodulation techniques such as TMS, tDCS, and iTBS temporarily adjust excitatory and inhibitory activity to prepare networks. Moreover, closed-loop feedback systems, such as BCI/BMI, VR, and neurofeedback, assist in optimizing oscillatory dynamics and corticomuscular coupling. Since there is no single “network fix”, this highlights the need for personalized, multimodal rehabilitation that adapts each approach to an individual’s specific connectivity profile.

Third, factors such as the amount of practice, quality of feedback, and timing between modalities have a more significant influence on outcomes than the specific technology brand. Patients showed clinically meaningful improvements after engaging in at least around 15 h of feedback-rich, task-specific practice over at least three weeks, whether it was facilitated by a robot, a therapist, or through self-guided practice.

Fourth, applying priming neuromodulation techniques early, such as cerebellar iTBS or bi-hemispheric tDCS, together with intensive training, consistently led to greater and more lasting network changes than using either method individually.

While methodological rigor and the quality of evidence are improving, variation in imaging techniques, cohort sizes, outcome metrics, and follow-up periods continues to hinder meta-analyses and accurate dose–response evaluations. To push the field forward, future studies should implement standardized protocols—such as combining resting-state fMRI with high-density EEG—and use uniform connectivity metrics along with multicenter randomized trials. These measures are essential to establish minimal effective “connectivity doses,” improve cost-efficiency, and facilitate the integration of network-based rehabilitation into everyday clinical practice.

### 5.1. Network–Behavior Summary and Practical Takeaway

Over 23 clinical studies (2015–2025), functional improvements most consistently correlate with: (i) increased interhemispheric SMN coupling (M1–M1; rs-fMRI Fisher-z), (ii) greater mu/alpha ERD and β-band CMC on EEG, and (iii) normalization of ALFF/ReHo within motor-related cortices. Multimodal protocols combining NIBS priming with feedback-intensive training—such as robotics, BCI/FES, and VR—exhibit the strongest connection between network modifications and behavior. In practice, tracking ERD, CMC, and M1–M1 alongside standardized behavioral measures can help optimize therapy dose and sequence.

### 5.2. Future Directions

The priorities involve: (1) incorporating effective-connectivity models like DCM and Granger into interventional trials; (2) standardizing measures of connectivity, variance, and data quality across different modalities; (3) exploring biomarker-based patient stratification to customize hybrid dosing; (4) assessing booster and home-based closed-loop treatment regimens; and (5) performing multicenter studies to determine the minimal effective “connectivity doses” and evaluate cost-efficiency.

## Figures and Tables

**Table 1:** Study selection process.

Search Terms	Filters	Inclusion Criteria	Exclusion Criteria
(“stroke rehabilitation” OR “post-stroke rehabilitation”) AND (“physical therapy” OR “occupational therapy” OR “neuromodulation” OR “neurofeedback” OR “infrared spectrography” OR “fMRI” OR “quantitative EEG”) AND (“functional disconnection” OR “functional connectivity” OR “resynchronization” OR “restoration of synchronicity”)	Date range: 2015–2025 Human studies English language	Humans with a stroke Original clinical or real-world trials Non-invasive stimulation, imaging, or feedback-based interventions Focus on disruptions or restoration of functional connectivity	Reviews, meta-analyses Animal studies No described intervention No mention of connectivity/disconnection

**Table 2:** Summary of Post-Stroke Functional Connectivity: Disruption, Stroke Rehabilitation Effects, and Relation to Motor, Sensory, and Cognitive Deficits. An expanded quantitative version with additional dose and statistical parameters is available in Supplementary Table S2.

Author & Year	Participants (Age, Sex, N)	Key Measures	Intervention	Connectivity Disruption	Effect of Intervention	Domain(s)
1. Yin [31]	34 (rTMS grp 16 M/2 F; no-stim grp 16 M/2 F; ≈57 y)	MoCA, VST, RBMT, MBI; ALFF, FC	10 Hz rTMS (L-DLPFC) + CACT (4 wk)	↓ DMN (MPFC–hippocampus)	↑ ALFF (L-MPFC), ↑ FC (R-MPFC → rACC) correlating with MoCA & VST improvements	Cognitive
2. Qin et al. [32]	49 (31 M/18 F; ≈59 y)	MAS, FMA-UE, MBI	1 Hz rTMS + 10 Hz rPMS (8 wk)	Reticulospinal ↑ excitability; ↓ inhibition	↑ ALFF (rSMA, rMFG, rCereb); ↓ ALFF (rPCG, lPrCG); improved spasticity & motor function	Motor
3. Middag-Van Spanje et al. [33]	22 (16 M/6 F; median ≈ 61 y)	SCT, CVDT, MLBT-d, SLBT, CBS, SNQ	10 Hz tACS + VST (6 wk)	Disrupted lateralized spatial attention	↑ Alpha synchronization; better contralesional detection; reduced visuospatial neglect	Cognitive/Spatial Attention
4. Chen et al. [34]	5 (3 M/2 F; 47–77 y)	UE-FM; rs-fMRI	Bihemispheric tDCS (1.5 mA) + PT/OT (2 wk)	↓ Interhemispheric motor connectivity	↑ Ipsilesional motor → contralesional premotor & precuneus connectivity → improved motor function	Motor
5. Sinha et al. [35]	23 (13 M/10 F; ≈62 y)	rs-fMRI; ARAT; 9-HPT; SIS; BI	EEG-BCI + FES (~15 sessions)	↓ Interhemispheric M1 connectivity	↑ M1–M1 and broader motor network rsFC; improved SIS (ADL, mobility)	Motor/ADL
6. Mekbib et al. [36]	8 stroke, 13 HC (≈57 y)	FM-UE; rs-fMRI	VR-LMT + conventional (1 h/day, 2 wk)	Bilateral M1 connectivity disrupted	↑ Interhemispheric M1 connectivity; correlated with FM-UE gains	Motor
7. Wittenberg et al. [37]	13 (12 M/1 F; 44–81 y)	TMS (MEP); MRI (FA, BOLD); FM; WMFT	Intensive robotic vs. conventional (6–12 wk)	Affected M1–PMAd connectivity changes	↑ M1–PMAd connectivity; correlated with improved motor outcomes	Motor
8. Fan et al. [38]	10 (8 M/2 F; ≈52 y)	FMA-UL; WMFT; FIM; rs-fMRI	Robot-assisted bilateral arm therapy (4 wk)	↓ Interhemispheric SMC connectivity	↑ M1–M1 rs-FC; improved FMA, WMFT, ADLs	Motor/ADL
9. Hu et al. [39]	19 stroke (14 M/5 F; ≈54 y), 11 HC	ALFF; ReHo; FC; FMA	MI-BCI ± tDCS (1 mA, 20 min; ~2 wk)	↓ SMN, disrupted DMN	MI-BCI only: ↑ ALFF in contralesional SMN; ↓ ALFF/ReHo in posterior DMN; better FMA	Motor/Cognitive
10. Wada et al. [40]	9 (6 M/3 F; ≈64 y)	EEG (ERD strength); physio data	DMB-based neurofeedback (14 d) + conventional	Impaired motor cortical connectivity → weaker ERD	22.9% ↑ ERD strength → reorganized motor pathways; improved spasticity	Motor
11. Sebastian et al. [41]	32 HC (42 y), 34 stroke (65 y)	EEG (BSI, LC); FMA; BBT; 9HPT	MI-BCI: VR avatar + FES (~25 sess)	Lateralization asymmetry (BSI, LC) → motor deficits	↑ Symmetry (BSI, LC); correlated with better FMA and function	Motor
12. Phang et al. [42]	10 (6 M/4 F; 39–80 y)	EEG (MRCP, FC); IMU; classification accuracy	Lower-limb motor tasks BCI (17 min)	PFCC disconnection	↑ PFCC strength hemiplegic side; marker of recovery	Motor/Sensorimotor Integration
13. Li et al. [43]	7 (5 HC, 2 stroke)	EEG–EMG (SPMI); isometric push/pull	GNN approach to EEG–EMG data	Traditional CMC inadequate	GNN: 88.9% accuracy; robust connectivity measure	Motor Intention Detection
14. Gangemi et al. [44]	30 (15 Exp/15 Ctrl; M = 20 F = 10; ≈58 y)	EEG (θ, α, β); clinical	Neurocognitive VR training (2D/3D)	(presumed) reduced α/β power	↑ α/β band power; enhanced connectivity; neural improvements	Cognitive/Motor
15. Ray et al. [45]	30 (18 M/12 F; ≈50 y)	cFMA; SMR; ERD (EEG)	BMI + physiotherapy (several wk)	Possible interhemispheric inhibition → ↓ α desync	↑ α desync ipsilesional; correlated with better motor recovery	Motor
16. Phang et al. [46]	11 (age ≈ 25 y)	EEG (frontoparietal corr.); BCI accuracy	Bipedal motor-prep BCI + neurofeedback	↓ Frontoparietal α → poor classification	Lowering α improved BCI performance; enhanced synchronization	Motor
17. Carino-Escobar et al. [47]	9 (5 M/4 F; 43–85 y)	EEG (α, β ERD/ERS); FMA-UE	BCI + robotic hand orthosis (4 wk)	β-band disruptions; nonhomologous hemispheres	↑ β power; correlated with motor recovery; cortical activation	Motor
18. Chen et al. [48]	46 (18–65 y)	BBS; TIS; balance tests; sEMG; fNIRS; FMA-LE; BI	Cerebellar vermis iTBS (3 wk) + PT	(implicit) vermis–cortical disruption	Hypothesized ↑ SMA excitability; better trunk/lower-limb activation	Motor/Balance
19. Ramos-Murguialday et al. [49]	28 (18–80 y)	cFMA; GAS; MAL; Ashworth; EMG; fMRI; LI	BMI + physiotherapy (1 h + 1 h/day, 4 wk)	(no long-term connectivity change)	Motor learning observed; EEG reorganization	Motor
20. Cheng et al. [50]	10 (4 M/6 F; ≈52 y)	fNIRS (OxyHb); sEMG; MSS	Robot-assisted hand therapy	(not specified)	↑ prefrontal & SMC OxyHb; improved muscle synergy/activation	Motor
21. Min Li et al. [51]	8 (M = 8; age ≈24.5 y)	Behavioral (accuracy, RT); P300 (ERP)	Exoskeleton hand + fingertip haptics	Disrupted motor–perception loop	↑ P300 amplitude; stronger M1/PM/S1 activation	Motor/Sensory Feedback
22. Ripollés et al. [52]	20 stroke (≈59 y), 14 Ctrl (≈56 y)	ARAT; APS; BBT; 9HPT; Barthel; neuropsych; fMRI	Music-supported therapy (4 wk)	↓ auditory–motor network (SMA–PRG, PAC–IFG)	↑ intrahemispheric connectivity (SMA–PRG, etc.); normalized network; gains in motor, sensory, some cognition	Motor/Sensory/Cognitive
23. Chen et al. [53]	72 (18–80 y; 4 groups)	MoCA; IADL; TCD (CBFV, PI, BHI)	CCT, tDCS, CACT, or CACT + tDCS (3 wk)	↓ DMN–FP correlation	CACT + tDCS: ↑ cerebrovascular reactivity; bilateral prefrontal excitability	Cognitive

Legend: Participants: M, Male; F, Female; y, years; wk, week(s). Clinical assessments: MoCA, Montreal Cognitive Assessment; VST, Victoria Stroop Test; RBMT, Rivermead Behavioral Memory Test; MBI, Modified Barthel Index; MAS, Modified Ashworth Scale; FMA-UE, Fugl-Meyer Assessment—Upper Extremity; UE-FM, Upper-Extremity Fugl-Meyer; ARAT, Action Research Arm Test; 9-HPT, Nine-Hole Peg Test; SCT, Star Cancellation Task; CVDT, Computerized Visual Detection Task; MLBT-d, Manual Line Bisection Task (difficult); SLBT, Standard Line Bisection Task; CBS, Catherine Bergego Scale; SNQ, Subjective Neglect Questionnaire; SIS, Stroke Impact Scale; BBT, Box & Block Test; FIM, Functional Independence Measure; IADL, Instrumental Activities of Daily Living; GAS, Goal Attainment Scale. Neurophysiology & imaging: EEG, electroencephalography (including ERD/ERS, MRCP, BSI, LC); ERP, event-related potential (e.g., P300); rs-fMRI, resting-state fMRI; fNIRS, functional near-infrared spectroscopy; TMS/MEP, transcranial magnetic stimulation/motor evoked potential; BOLD, blood-oxygenation-level-dependent signal; ALFF, amplitude of low-frequency fluctuations; ReHo, regional homogeneity; FC, functional connectivity; CMC, cortico-muscular coherence; FA, fractional anisotropy; CBFV, cerebral blood-flow velocity; PI, pulsatility index; BHI, breath-holding index; LI, laterality index; sEMG, surface electromyography. Interventions & technologies: rTMS, repetitive transcranial magnetic stimulation (10 Hz, 1 Hz); tDCS, transcranial direct current stimulation; tACS, transcranial alternating current stimulation; iTBS, intermittent theta-burst stimulation; rPMS, repetitive peripheral magnetic stimulation; BCI/BMI, brain–computer (or-machine) interface; FES, functional electrical stimulation; VR/VR-LMT, virtual reality (limb movement training); robot-assisted therapy; CACT, computer-assisted cognitive training; PT/OT, physical/occupational therapy; neurofeedback. ↑ Increased; ↓ Decreased. Brain networks & regions: DMN, default mode network; SMN, sensorimotor network; Salience Network; PFCC, parieto-frontocentral connectivity; Motor Network (M1/PM/S1); MPFC, medial prefrontal cortex; rACC, right anterior cingulate cortex; SMA, supplementary motor area.

**Table 3:** Descriptive Statistics Across Studies (Dose & Sample Characteristics).

Descriptor	Median [IQR]	Notes
Per-study N	NR	Report median [IQR] once all rows are fully populated (many reports do not provide exact Ns by arm in our dataset).
Minutes per session	120 [29,53–85,94–120]	Derived from reported values where available (e.g., BMI + PT ≈ 120 min; VR ≈ 60 min).
Sessions per week	5 [5]	Typical frequency in structured programs (5 sessions per week where reported).
Total sessions	20 [10–25]	Based on median across trials (e.g., VR ≈ 10, BMI ≈ 20, robotics ≈ 24).
Protocol duration (weeks)	NR	To be computed from total sessions and frequency where both parameters are available.

Values computed only from studies reporting explicit numeric data; ‘NR’ indicates no reporting or non-comparable formats. The accompanying Excel supplement preserves all cell-level entries for transparency.

**Table 4:** Network-Guided Timing & Dose (Clinically Actionable).

Patient Phase	Primary Disruption	Recommended Pairing	Typical Dose Window	Biomarkers to Monitor	Expected Change
Subacute (<3 mo), subcortical or M1-adjacent	SMN (M1–M1), parietal–premotor	tDCS/iTBS (priming) → robot-assisted bilateral, task-specific practice	Priming immediately before practice; ≥15 h over ≥3 wk	↑ ERD (mu/alpha), ↑ M1–M1 rsFC; ↑ CMC	Reduced interhemispheric imbalance; ↑ FMA-UE/WMFT
Chronic (>6 mo), persistent imbalance	SMN timing & cortico-muscular coupling	EEG-BCI (ERD-triggered) + FES/robotic orthosis (closed-loop)	45–60 min, 3–5×/wk, ≥3–6 wk	↑ ERD; ↑ CMC; normalization of LI	↑ dexterity/strength; ADL gains
Cognitive/executive comorbidities	FPN–DMN–Salience control	VR/AOT + CACT ± prefrontal NIBS	30–45 min, 3×/wk, 3–6 wk	↑ alpha (occipital), ↑ beta (frontal), ↑ FP coupling	↑ attention/executive function; transfer to motor planning
Balance/posture deficits	Cerebello–thalamo–cortical	Cerebellar NIBS (iTBS or tACS) + balance/locomotor practice	Short priming blocks preceding training	↑ SMA excitability proxies; ↑ cerebello–cortical coupling	↑ trunk control/BBS; gait improvements

Abbreviations: mo, months; wk, weeks; SMN, sensorimotor network; FPN, frontoparietal network; DMN, default mode network; AOT, action observation training; CACT, computer-assisted cognitive training; tDCS, transcranial direct current stimulation; iTBS, intermittent theta-burst stimulation; tACS, transcranial alternating current stimulation; BCI, brain–computer interface; FES, functional electrical stimulation; ↑, Increased; ERD, event-related desynchronization; CMC, cortico-muscular coherence; LI, laterality index; FMA-UE, Fugl-Meyer Assessment –Upper Extremity; WMFT, Wolf Motor Function Test; BBS, Berg Balance Scale; ADL, activities of daily living.

**Table 5:** Connectivity Metrics & Normalization (Definitions, Units, Caveats).

Metric	Definition	Functional Meaning	Normalization/Units	Strengths	Caveats
rs-fMRI correlation	Fisher-z Pearson correlation between ROI time series	Network coupling at rest	Fisher-z; motion-scrubbed BOLD	Reproducible; network-level	Motion/physiology sensitive; hemodynamic
ERD/ERS (EEG)	% change from baseline in band power (mu/alpha, beta)	Sensorimotor engagement; recovery prediction	% from baseline; referenced	High temporal resolution	Volume conduction; reference effects
PLV/PLI/wPLI	Phase (lag) synchrony between regions	Functional coupling; wPLI reduces volume conduction	0–1; z-scored	Robust synchrony	Not causal; noise sensitive
Coherence	Linear frequency-domain coupling	Spectral coupling	0–1; sometimes Fisher-z	Simple, common	Stationarity; volume conduction
CMC	Cortico-muscular coherence (EEG–EMG)	Brain–muscle communication	Magnitude or Fisher-z	Direct motor relevance	Requires clean EMG
ALFF/ReHo	Amplitude of low-freq fluctuations/local homogeneity	Regional activity/local synchrony	zALFF/zReHo	Easy to compute	Indirect; modality noise
BSI/LC	Brain symmetry index/laterality coefficient	Hemispheric balance	Unitless indices	Simple asymmetry	Coarse; hides network specifics

Abbreviations: rs-fMRI, resting-state functional MRI; ROI, region of interest; BOLD, blood-oxygen-level-dependent; ERD/ERS, event-related desynchronization/synchronization; PLV/PLI/wPLI, phase/weighted phase lag index; CMC, cortico-muscular coherence; ALFF, amplitude of low-frequency fluctuations; ReHo, regional homogeneity; BSI, brain symmetry index; LC, laterality coefficient.

## Data Availability

No new data were created or analyzed in this study. Data sharing is not applicable to this article.

## Supplementary Materials

The following supporting information can be downloaded at: https://www.mdpi.com/article/10.3390/brainsci15111217/s1, Table S1: RoB 2 (RCTs); Table S2: A complete data-extraction table for all included interventional/observational studies summarized in the scoping review (Table 2). Reference [34] is cited in Supplementary Materials.

## Abbreviations

ADL, activities of daily living; ALFF, amplitude of low-frequency fluctuations; AOT, action-observation training; ARAT, Action Research Arm Test; BBI, (remove if unused); BBS, Berg Balance Scale; BCI, brain–computer interface; BHI, breath-holding index; BI, Barthel Index; BMI, brain–machine interface; BOLD, blood-oxygen-level-dependent; BSI, brain symmetry index; CACT, computer-assisted cognitive training; CBFV, cerebral blood-flow velocity; CIs, confidence intervals; CMC, cortico-muscular coherence; CVDT, Computerized Visual Detection Task; DAN, dorsal attention network; DMN, default mode network; EEG, electroencephalography; EMG, electromyography; ERD/ERS, event-related desynchronization/synchronization; FES, functional electrical stimulation; FIM, Functional Independence Measure; fMRI, functional magnetic resonance imaging; fNIRS, functional near-infrared spectroscopy; FP, frontoparietal; FPN, frontoparietal network; GAS, Goal Attainment Scale; GNN, graph neural network; HC, healthy control; IADL, instrumental activities of daily living; iTBS, intermittent theta-burst stimulation; LC, laterality coefficient; LI, laterality index; MAL, Motor Activity Log; MAS, Modified Ashworth Scale; MEP, motor-evoked potential; MI, motor imagery; MoCA, Montreal Cognitive Assessment; MRCP, movement-related cortical potential; NR, not reported; OxyHb, oxygenated hemoglobin; PAC, primary auditory cortex; PFCC, parieto-frontocentral connectivity; PLI/PLV/wPLI, (weighted) phase-lag index/phase-locking value; PRG, precentral gyrus; PRISMA-ScR, Preferred Reporting Items for Systematic reviews and Meta-Analyses extension for Scoping Reviews; PT/OT, physical/occupational therapy; ReHo, regional homogeneity; rPMS, repetitive peripheral magnetic stimulation; rs-fMRI, resting-state fMRI; rTMS, repetitive transcranial magnetic stimulation; S1, primary somatosensory cortex; SMA, supplementary motor area; SMC, sensorimotor cortex; SMN, sensorimotor network; SMR, sensorimotor rhythm; TCD, transcranial Doppler; TMS, transcranial magnetic stimulation; UE-FM (or FMA-UE), Upper-Extremity Fugl-Meyer Assessment; VST, Victoria Stroop Test; VR, virtual reality; WMFT, Wolf Motor Function Test.
